# Histopathological Pulmonary Lesions in 1st-Day Newborn Piglets Derived from PRRSV-1 MLV Vaccinated Sows at the Last Stage of Gestation

**DOI:** 10.3390/life13071609

**Published:** 2023-07-23

**Authors:** Georgios I. Papakonstantinou, Dimitra Psalla, Aris Pourlis, Ioanna Stylianaki, Labrini V. Athanasiou, Eleni Tzika, Eleftherios Meletis, Polychronis Kostoulas, George Maragkakis, Georgios Christodoulopoulos, Nikolaos Papaioannou, Vasileios G. Papatsiros

**Affiliations:** 1 Clinic of Medicine, Faculty of Veterinary Medicine, School of Health Sciences, University of Thessaly, 43100 Karditsa, Greece; geopapak@vet.uth.gr (G.I.P.); lathan@vet.uth.gr (L.V.A.); gmaragkakis@uth.gr (G.M.); 2 Laboratory of Pathology, Faculty of Health Sciences, School of Veterinary Medicine, Aristotle University of Thessaloniki, 54124 Thessaloniki, Greece; dpsalla@vet.auth.gr (D.P.); stylioan@vet.auth.gr (I.S.); nikpap@vet.auth.gr (N.P.); 3 Laboratory of Anatomy, Histology & Embryology, Veterinary School, University of Thessaly, 43100 Karditsa, Greece; apourlis@vet.uth.gr; 4 Farm Animals Clinic, School of Veterinary Medicine, Aristotle University of Thessaloniki, 54627 Thessaloniki, Greece; eltzika@vet.auth.gr; 5 Laboratory of Epidemiology & Artificial Intelligence, Faculty of Public Health, School of Health Sciences, University of Thessaly, 43100 Karditsa, Greece; elmeletis@uth.gr (E.M.); pkost@uth.gr (P.K.); 6 Department of Animal Science, Agricultural University of Athens, 75 Iera Odos Street, Votanikos, 11855 Athens, Greece; gc@aua.gr

**Keywords:** piglet, MLV vaccines, safety, lung, histopathological lesions, scanning electron microscopy

## Abstract

Modified live virus (MLV) vaccines for the control of porcine respiratory and reproductive syndrome virus (PRRSV) have been associated with the vertical and horizontal transmission of vaccine viruses. The present study aimed to describe pathological lung lesions in piglets born by gilts vaccinated with PRRSV-1 MLV. In total, 25 gilts were vaccinated at late gestation (100th day) and were divided into five groups according to the different vaccines (Vac) used: no vaccine—control group, Vac-1—strain DV, Vac-2—strain VP-046 BIS, Vac-3—strain 94881, Vac-4—strain 96V198. Within the first 0–9 h of the farrowing, blood samples were collected from all newborn piglets and lung samples were exanimated grossly, histopathologically and with scanning electron microscopy. PRRSV (RT-PCR-positive) and antibodies were detected in the serum of piglets from gilts vaccinated with Vac-2. In these piglets, moderate to severe interstitial pneumonia with thickened alveolar septa was noticed. Type II pneumocyte hyperplasia was also observed. The rest of the trial piglets showed unremarkable lung lesions. Phylogenetic analysis revealed the 98.7% similarity of the PRRSV field strain (GR 2019-1) to the PRRS MLV vaccine strain VP-046 BIS. In conclusion, the Vac-2 PRRSV vaccine strain can act as an infectious strain when vaccination is administrated at late gestation, causing lung lesions.

## 1. Introduction

The clinical forms of porcine reproductive and respiratory syndrome (PRRS) include reproductive failures in breeding stock (abortions, mummified fetuses, and weak-born piglets) and respiratory disease in young pigs [1,2,3]. PRRS is caused by an RNA virus referred to as PRRS virus (PRRSV), which is initially distinguished as genotypes PRRSV-1 (European) and PRRSV-2 (North American) [4]. According to the recent classification of the International Committee on Taxonomy of Viruses [5], previous genotypes are now considered to constitute two distinct species named *Betaarterivirus suid 1* and *Betaarterivirus suid 2* and classified within two different subgenera, *Eurpobartevirus* and *Ampobartevirus*, in the genus *Betaarterivirus* (subfamily *Variarterivirinae*, family *Arteriviridae*, suborder *Arnidovirinae*, order *Nidovirales*) [6].

During PRRSV infection, apoptosis is induced in host cells both in vitro and in vivo. The induction of apoptosis in host cells is an important cellular mechanism contributing to the pathogenesis of PRRSV. After the exposure of pigs to the virus (via the respiratory tract and oral ingestion), it replicates mainly in porcine macrophages and especially in pulmonary alveolar macrophages [7,8]. As a result of virus replication, pulmonary alveolar macrophage function is impaired and the infiltration of inflammatory cells occurs, leading to interstitial pneumonia, which is the most common lesion type in PRRS infection [9,10,11]. In addition, typical microscopic lesions caused by PRRS in the lungs include hypertrophy and hyperplasia of type-2 pneumocytes [9,10]. Finally, the virus spreads rapidly throughout the body via the lymphatic–hematic route and causes various lesions in PRRS-infected tissue, which is probably the most important pathogenic event leading to increased susceptibility to secondary infection and even to death [12,13].

Management, monitoring, and vaccination are the pillars on which a PRRS control plan is usually based. Monitoring and vaccination are the key components of most PRRS disease control strategies. In PRRS surveillance, the serum is the reference sample for the determination of the pathogen. Furthermore, histopathological studies can provide valuable information on the pathogenetic impact of the virus, by identifying lesions that occur in PRRSV-infected tissue. Electron microscopy is also a useful tool for the detection of the virus during an outbreak [14]. Regarding PRRS vaccination, several commercial vaccines are available on the market and are based on either a modified live virus (MLV) or killed virus (KV). Unfortunately, the existing vaccines are largely ineffective because they cannot provide complete protection. Nowadays, MLV vaccines are widely used in practice because they elicit a stronger and more complete immune response compared to KV vaccines [15,16,17,18,19]. Although they offer good virological and clinical protection against a homologous challenge, they induce only partial immunity against heterologous challenges. Nevertheless, they are effective to reduce abortions and the mortality of newborn piglets and improve reproductive parameters in PRRSV-infected sows [20,21,22].

Many studies have raised concerns about the safety of MLV vaccines because of the risk of the vertical and horizontal transmission of the vaccine virus. In particular, PRRSV MLV-vaccinated pigs may develop viremia due to the MLV virus and transmit the virus to other naïve animals [22,23,24]. Previous studies reported possible congenital infections in the last stage of gestation in sows [1,15,25]. However, the efficiency of MLV vaccines under field conditions depends not only on the biological properties of the MLV vaccine but also on the vaccination protocol used. Currently, the main routine protocols used are based on initial double vaccination in gilts before insemination and subsequent recall vaccination in sows, using either the 6–60 strategy (6 days after farrowing and 60 days of gestation) or the “mass/blanket” vaccination program (3–4 vaccinations/year, all animals at the same time). Mass vaccination is a common routine vaccination practice in many countries, including Greece. However, critical issues such as non-observance of this vaccination protocol could lead to PRRSV infection. The most common mistakes made include the vaccination of sows during the last stage of gestation (>95th day), inducing a potential fetal infection, or the use of different commercial MLV vaccines in every booster vaccination [17,22,26,27,28]. Another crucial point is the heterogenicity of the vaccine virus of the various commercial PRRSV MLV vaccines [29,30,31]. Unfortunately, few studies document the advantages and disadvantages of each vaccination strategy.

Under field conditions and based on our experience, many pig farms apply PRRSV MLV vaccinations at the late stage of gestation, without complying with the appropriate restrictions, and, consequently, they are at risk of potential viral problems related to mass MLV vaccination. The literature data are limited about the possible induced pulmonary lesions in newborn piglets derived from vaccinated sows with a heterologous PRRSV MLV vaccine at the last stage of gestation. PRRSV MLV vaccine strains can act as infectious strains when the sows are vaccinated at late gestation, resulting in the congenital infection of piglets during their uterine life, affecting their immunity [28].

The present study aimed to investigate the ability of the PRRSV MLV vaccine virus, at mass vaccination at the late stage of gestation, to be transmitted to fetuses, and subsequently to describe the possible gross, histopathological, and scanning electron microscopy (SEM) findings associated with PRRS-induced lung lesions.

## 2. Materials and Methods

### 2.1. Ethics

The present study was carried out under the appropriate approval (number 65/26 February 2019) of the Institutional Animal Use Ethics Committee (Faculty of Veterinary Medicine, University of Thessaly, Greece).

### 2.2. Trial Farm

The present study was conducted on a commercial farrow-to-finish pig farm located in South Greece (Aspra Spitia, Archaia Olympia, Ilia—37°39′00.5″ N 21°37′34.4″ E), with a capacity of approximately 400 sows of the same genetic background (commercial hybrids of Large White × Landrace). A grandparent nucleus of the sows was kept on the farm to produce its replacement gilts. The farm facilities included an artificial insemination laboratory as well as a feed mill for the self-prepared feed based on corn/barley/wheat/soya meal. The ad libitum consumption of drinking water was allowed in the farm and the temperature and humidity of the housing facilities were controlled by an automated control system.

### 2.3. Farm’s History

The history of the trial farm included previous severe PRRSV outbreaks, characterized by reproductive failure (increased abortions and return-to-estrus rates, increased farrowing with mummified and stillborn piglets) and respiratory distress in weaning piglets. A vaccination program against PRRSV was applied for more than a decade, including the initial vaccination of the gilts twice (180th day and 210th day of age) before their first insemination and the mass vaccination of the sows 3–4 times/year, applying a commercial PRRSV MLV vaccine (Porcillis PRRS, MSD Animal Health).

During the field trial period, no PRRSV outbreaks were noticed, as all blood samples from all age groups (gilts, sows during lactation and dry period, weaners, growers, and fatteners) were reverse transcription polymerase chain reaction (RT-PCR)-negative for PRRSV. These results indicated also that the routine vaccination program against PRRSV successfully controlled the circulation of the virus in the trial farm.

### 2.4. Trial Design and Sampling

**Figure 1:**
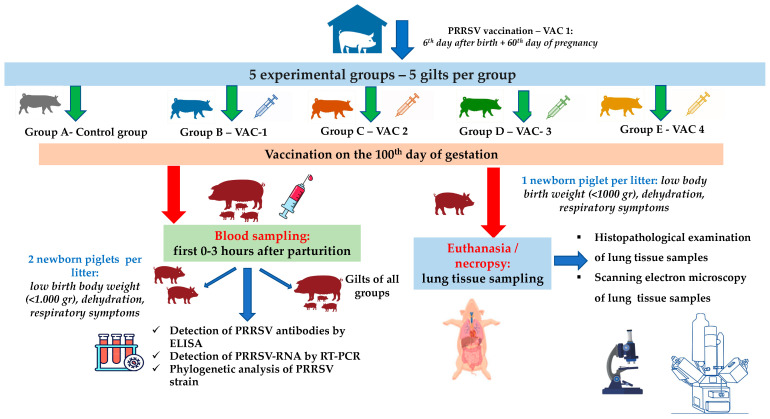
A flowchart of the trial (groups, study design, sampling, laboratory examinations).

In total, 25 gilts that were RT-PCR-negative for PRRSV were equally divided into 5 groups (5 gilts per group, Figure 1). The selected gilts were of the same genetic background (commercial hybrids of Large White × Landrace), with a mean age of 174.5 ± 5.6 days and 98.8 ± 10.6 kg body weight (BW). All gilts were vaccinated with a commercial vaccine (Porcillis PRRS, MSD Animal Health, strain DV) at the 180th and 210th days of age, plus the 60th day of their first gestation.

Previous studies reported that PRRSV can cross the placenta after the 95th day of gestation [25,32,33]. For this reason, the trial design included an additional vaccination on the 100th day of gestation with the following commercial PRRSV-1 MLV vaccines: (a) Vaccine One (Vac-1)—Porcillis PRRS (MSD Animal Health, strain DV); (b) Vaccine Two (Vac-2)—UniStrain PRRS (Hipra, strain VP-046 BIS); (c) Vaccine Three (Vac-3)—ReproSyc PRRS (Boehringer Ingelheim Vetmedica, strain 94881); and (d) Vaccine Four (Vac-4)—Suvaxyn PRRS (Zoetis, strain 96V198) (Table 1). All animals of the five groups were housed in different barns with separate air spaces, and they were housed under similar conditions (climate, ventilation, temperature, and air humidity) controlled by an automated control system.

Blood sampling from all gilts was conducted within the first 0–3 h of their parturition, using S-Monovette^®^ 9 mL (Sarstedt AG & Co. KG, Numbrecht, Germany) and disposable 14G X 3-1/414, 2.1 × 80 mm needles (Jørgen Kruuse A/S, Langeskov, Denmark) (Figure 1). Moreover, blood sampling was conducted from two piglets/litter/group (total 50 samples) that displayed a poor clinical condition in the first few hours after birth and before the consumption of colostrum (low birth BW < 1.000 g, dehydration, presence of respiratory symptoms—Figure 1). For the collection of blood samples from piglets, S-Monovette^®^ 9 mL (Sarstedt AG & Co. KG, Numbrecht, Germany) and disposable 21Gx1½, 0.8 × 40 mm needles (Jørgen Kruuse A/S, Langeskov, Denmark) were used (Figure 1). Serum was collected from all blood samples after centrifugation (10 min at 3000× *g*) and was stored at −80 °C for further laboratory analysis.

Finally, one piglet/litter/group with the poorest performance and before the consumption of colostrum in each litter was euthanized by IV administration of 0.2 mL/kg pentobarbital (Exagon^®^ Richter Pharma AG, Wels, Austria) [34]. Lunney et al. (2016) [13] reported that the initial PRRSV infection is followed by the virus’ replication, mainly in the lungs. Therefore, the lungs were exanimated for macroscopic lesions and lung tissue samples were collected for histopathological and SEM examinations.

### 2.5. Records

All gilts were daily and individually examined by the same veterinarian, from the day before vaccination until the first 24 h after farrowing. A clinical observation score system was applied, including an assessment of behavior, respiratory signs, and fever. Systemic and local adverse reactions at the site of injection were examined daily (e.g., redness, swelling, heat, pain) from the day of vaccination until farrowing (one time per h and for 4 h after vaccination). Moreover, the reproductive parameters of all vaccinated gilts were recorded, including the duration of gestation, abortion and return-to-estrus rates, and litter characteristics (number of healthy, live-born, dead-born, weak-born, mummies, and splay-leg piglets). At birth, the piglets were physically examined every day, starting from the day of birth until the weaning day. A clinical observation score system was applied, including the evaluation of behavior and digestive and respiratory signs. Piglets with poor clinical performance were also recorded.

### 2.6. Laboratory Examinations

#### 2.6.1. ELISA—PRRSV-Specific Antibodies (PRRSV-Abs)

All serum samples were examined for PRRSV-Abs, using commercial ELISA kits (Civtest Suis PRRS E/S^®^ Plus and Civtest Suis A/S^®^ Kits—Laboratorios Hipra, Girona, Spain). These kits provided the ability to detect circulating PRRSV-Abs against both PRRSV-1 and PRRSV-2. The analysis procedures and the interpretation of the results were conducted according to the manufacturer’s guidelines. Positive samples were considered as samples with a relative index (expressed as a percentage) greater than 20 [35].

#### 2.6.2. Detection of PRRSV-RNA by RT-PCR and ORF5 Sequencing

Viral RNA was purified from all serum samples using the RNeasy Mini Kit (Qiagen, Hilden, Germany) in an automatic robot (QIAcube, Qiagen), according to the manufacturer’s instructions, as previously described [28]. Finally, a phylogenetic analysis was performed on positively tested blood samples. The entire procedure was conducted in the Laboratorios Hipra (Spain).

#### 2.6.3. Histopathology

Euthanized piglets were necropsied to evaluate possible gross lung lesions. Moreover, lung tissue samples were collected for histopathological examinations and were fixed in 10% buffered formalin for 48 to 72 h. Following this, they were dehydrated by immersion in a series of alcohol solutions and embedded in paraffin routinely (Paraplast Plus^®^, Kendall, UK). Dewaxed 3–5-μm-thick sections were obtained and stained with hematoxylin and eosin (H&E) for histopathological examination.

#### 2.6.4. Scanning Electron Microscopy (SEM)

Tissue samples from the lungs were fixed in 2.5% (*w*/*v*) glutaraldehyde solution in a sodium cacodylate buffer (0.1 mol/L pH 7.3) and stored in the fixative at 4 °C until processed for SEM. The specimens were dehydrated in an acetone series, dried in a critical point drier using carbon dioxide (CO_2_), mounted on the specimen holder, coated with gold with a thickness of 14 nm, and observed under an accelerated voltage of 10.0 KV at a short working distance, in a Cambridge Stereoscan 360° SEM.

### 2.7. Statistical Analysis

The minimum population size was estimated by performing sample size estimation with the G*Power 3.1 software [35], ensuring that the study power was higher than 95%. Data associated with the reproductive characteristics of one hundred sows after parturition were available. The objective of the analysis was to apply a linear regression model that predicted the antibody level response, given covariate information, which is a statistically significant variable. The explanatory variables inspected as potential predictors of the Ab level in this study were the following: (i) PCR sample binary result; (ii) time interval between piglet birth and sample collection, as a categorical variable [0–3 h: 0, 3–6 h: 1, >6 h: 2].

All candidate variables were initially screened one-by-one, with a significance level of 0.25. Variables with *p* < 0.25 were then offered to the full model, which was reduced by backward elimination until only significant (*p* < 0.05) variables remained. The slope coefficient was the reported estimate, and 95% confidence intervals (CIs) for each coefficient were constructed. The coefficient reflected how the response variable changed for any change in this predictor, given that all the other predictors included in the model were held constant.

Most potential risk factors are categorical explanatory variables with more than two levels. Given the 0.05 significance level set in the full model (0.25 at the initial screening), if one level of the risk factor had a *p*-value above the significance level, while the *p*-values for the rest of the levels were less than the significance level, the predictor was considered statistically significant.

## 3. Results

### 3.1. Clinical Observations

No systemic or adverse reactions were noticed in vaccinated pregnant gilts. The clinical observations of the piglets per group are shown in Table 1.

### 3.2. Laboratory Results

#### 3.2.1. PRRSV-Abs

All samples from the trial gilts, as well as 18% (9/50) of the samples from selected piglets (5 piglets from group C, 2 piglets from group D, and 2 piglets from group E), were positive in the ELISA test. PRRSV-Abs were detected in both RT-PCR-negative and RT-PCR-positive piglets.

#### 3.2.2. Detection of PRRSV-RNA

**Figure 2:**
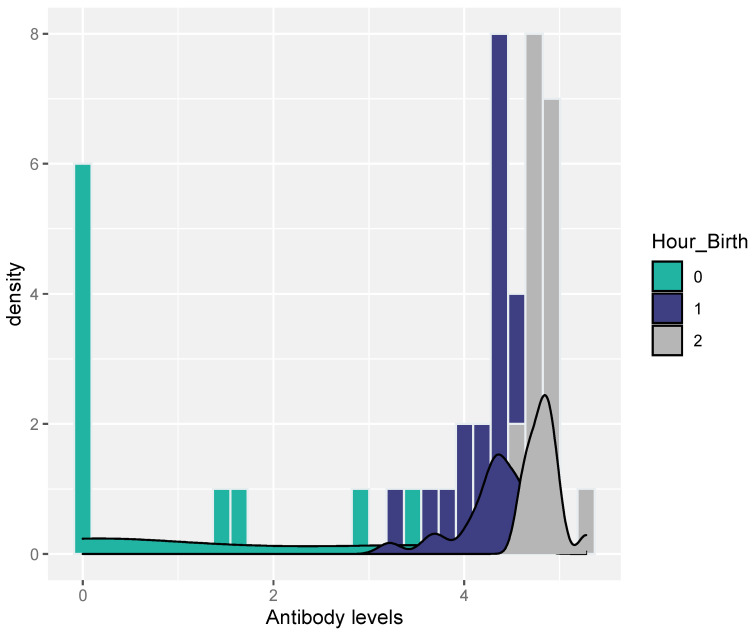
Antibody distribution for each level of Hour_birth [0 → 0–3 h, 1 → 3–6 h, 2 → >6 h].

Serum samples from all study gilts were RT-PCR-negative for PRRSV. A small percentage of the samples (20% = 10/50) from the selected piglets were RT-PCR-positive for PRRSV (piglets born in group C). PRRSV-Abs were also detected in the samples of the selected piglets that were collected immediately after birth and before the consumption of colostrum (0–3 h), as shown in Table 2 and Figure 2. However, samples were also taken a few hours after birth (3–6 h) and several hours after birth (6–9 h) from piglets derived from gilts that were farrowed during the night and escaped early morning observation sampling.

#### 3.2.3. Phylogenetic Analysis of Isolated PRRSV Strain

The phylogenetic analysis revealed that the isolated PRRSV field strain (GR 2019-1) had 98.7% similarity to the Vac-2 vaccine strain (VP-046 BIS).

### 3.3. Pulmonary Lesions

#### 3.3.1. Gross Pathology

**Figure 3:**
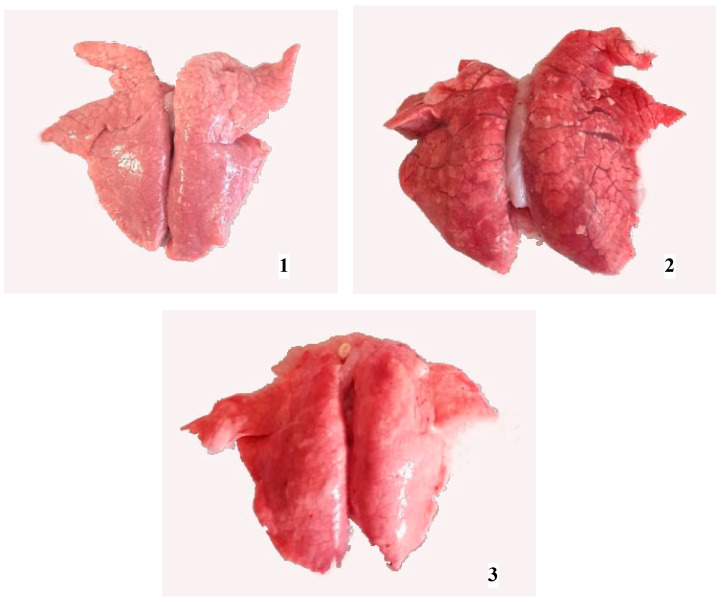
Lungs obtained from newborn piglets: diffuse discoloration and moderate consolidation, piglet born in group C (**1**); mild interstitial edema and multifocal discoloration, piglet born in group D (**2**); no remarkable changes, piglet born in group A (**3**).

The necropsy of piglets born in group A revealed no remarkable gross lung lesions. Piglets from group C presented either diffuse discoloration and moderate consolidation or interstitial edema and mild consolidation accompanied by scattered hemorrhages (Figure 3). As for the rest of the trial piglets (groups B, D, and E), they showed only mild edema and hyperemia in their lungs.

#### 3.3.2. Histopathological Lesions

**Figure 4:**
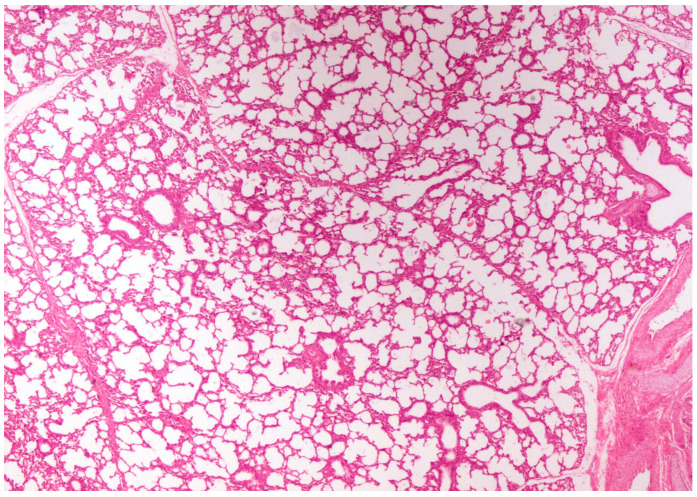
Normal lung architecture was obtained from newborn piglets born in group A. H&E staining (40×).

**Figure 5:**
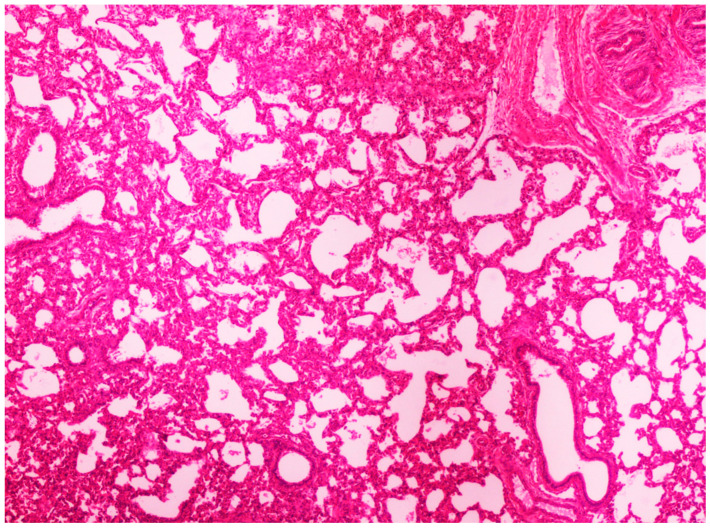
Thickening of alveolar septa due to infiltration by inflammatory cells. Lungs obtained from newborn piglet born in group C. H&E staining (100×).

**Figure 6:**
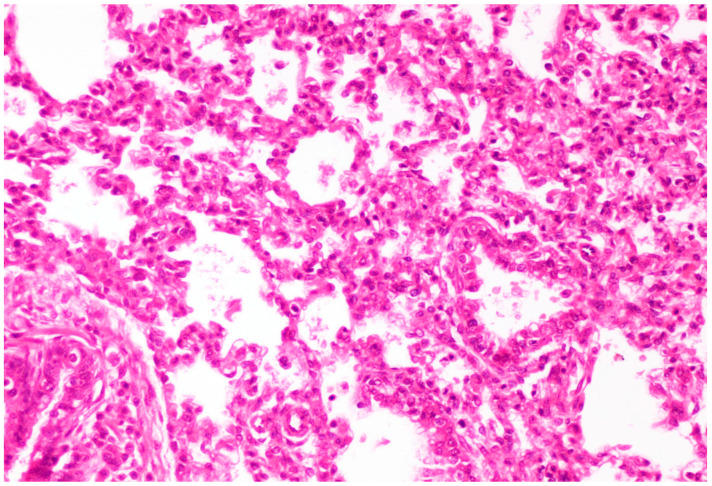
Thickening of alveolar septa due to infiltration by mainly lymphocytes and plasma cells. Scattered alveolar macrophages are noticed in the alveolar lumens. Lungs obtained from newborn piglet born in group C. H&E staining (200×).

**Figure 7:**
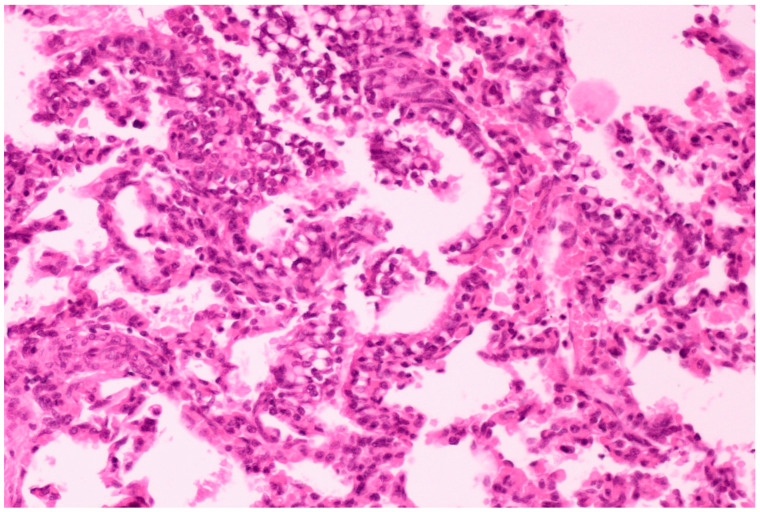
Thickening of alveolar septa due to infiltration by mainly lymphocytes and plasma cells. Scattered alveolar macrophages are noticed in the alveolar lumens, accompanied by hyperplasia of bronchiolar epithelium. Lungs obtained from newborn piglet born in group C. H&E staining (200×).

**Figure 8:**
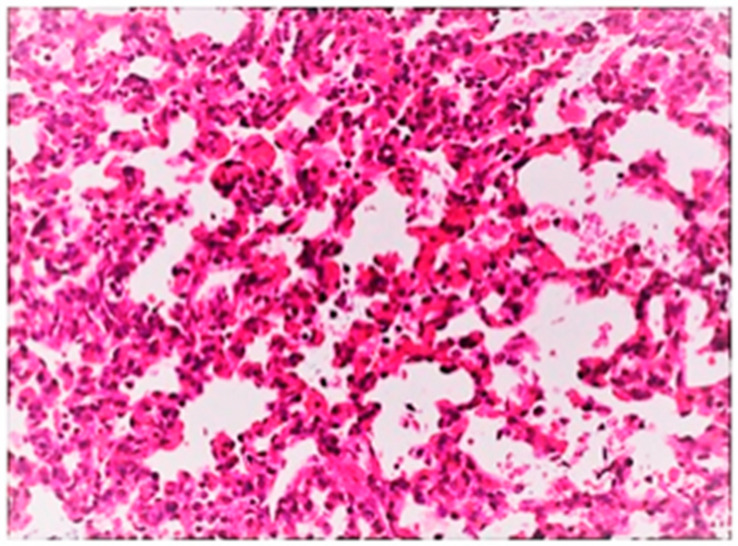
Hyperemia and thickening of alveolar septa due to infiltration by mainly lymphocytes and plasma cells. Lungs obtained from newborn piglet born in group D. H&E staining (200×).

On histological examination, the lungs of all control piglets did not exhibit any microscopic lesions (Figure 4). Piglets born in group C presented interstitial edema, mild interstitial hyperemia, and diffuse thickening of the alveolar septa due to infiltration by moderate to large numbers of lymphocytes and plasma cells and fewer histiocytes. Μany alveolar spaces contained proteinaceous fluid, admixed with alveolar macrophages and/or a few neutrophils. Multifocal bronchi and bronchioli underwent epithelial hyperplasia (Figure 5, Figure 6 and Figure 7). Piglets born in groups B, D, and E exhibited the abovementioned histopathological lesions of a milder degree (Figure 8).

#### 3.3.3. Scanning Electron Microscopy (SEM) Findings

**Figure 9:**
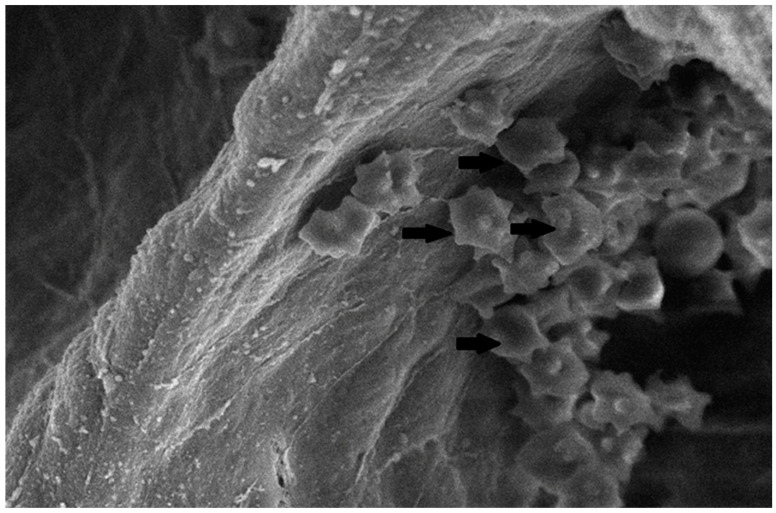
Pulmonary alveoli from RT-PCR (+) newborn piglet: accumulation of crenated erythrocytes (arrows) over the alveolar wall. SEM (2500×).

**Figure 10:**
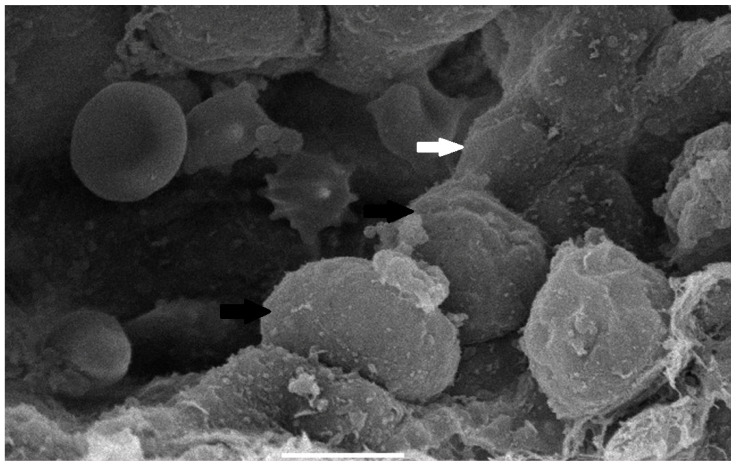
Pulmonary alveoli from RT-PCR (+) newborn piglet: accumulation of leukocytes, presumably lymphocytes (black arrows), resulted in a thickened alveolar wall (white arrow). SEM (4300×).

**Figure 11:**
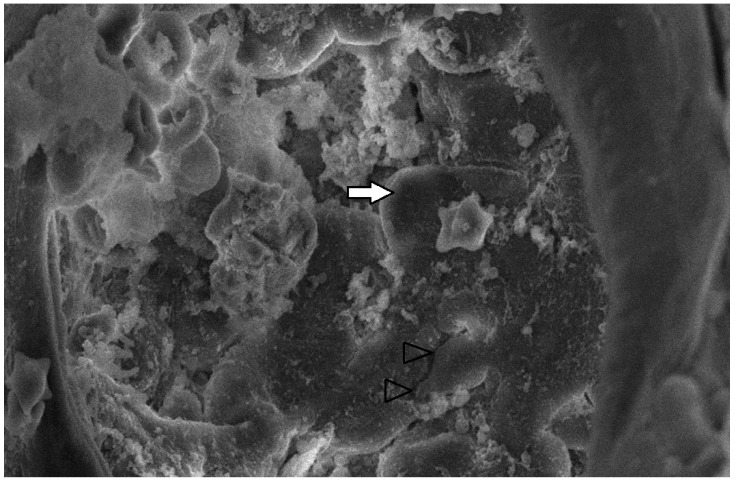
Pulmonary alveoli from PRRSV RT-PCR (+) newborn piglet: alveolar lumen is filled with proteinaceous and cellular exudate, including erythrocytes, presumably macrophages (white arrow). Separation between type I pneumocytes, implying tight junctions lost, is indicated by open arrowheads. SEM (2500×).

**Figure 12:**
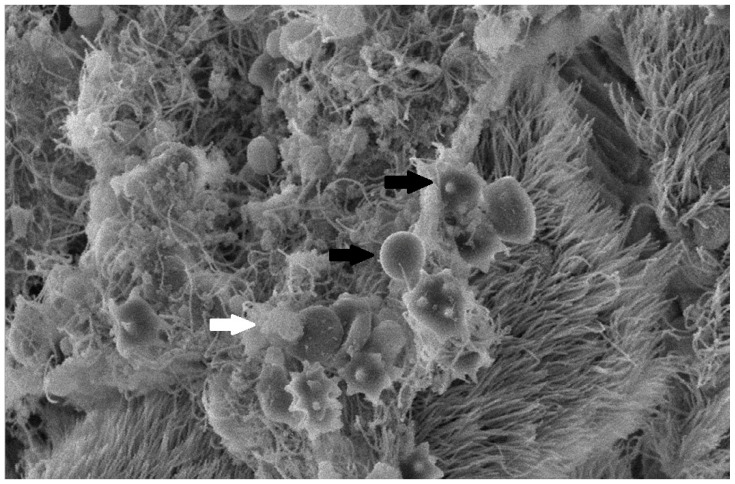
Pulmonary bronchi from RT-PCR (+) newborn piglet: accumulation of proteinaceous and cellular exudate (white arrow), with crenated erythrocytes (black arrow). SEM (2500×).

**Figure 13:**
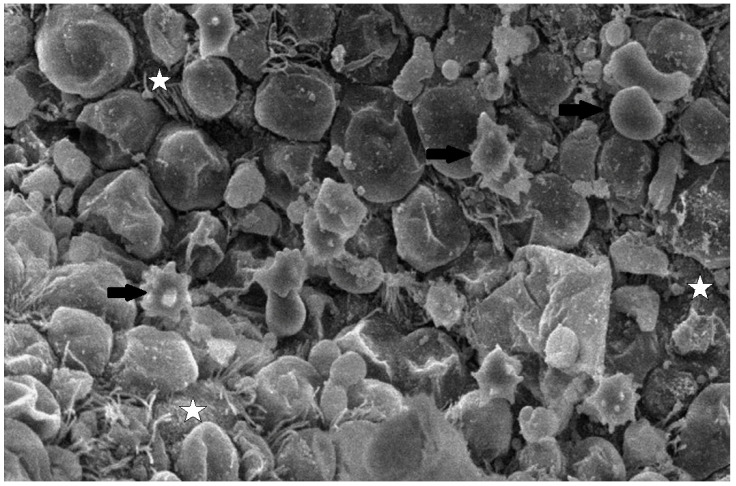
Pulmonary bronchi from RT-PCR (+) newborn piglet: accumulation of inflammatory exudate with crenated erythrocytes (black arrows), foci with apical cytoplasmic blebbing, and loss of cilia (star). SEM (2500×).

The collected lung tissue of PRRSV RT-PCR (+) newborn piglets was examined under SEM. Specifically, the exudation of erythrocytes and presumably leukocytes (lymphocytes and macrophages) in the pulmonary alveoli was observed (Figure 9 and Figure 10). In addition, a small number of presumably type II pneumocytes, which supported the histological findings of hyperplasia, were also recorded (Figure 11). Erythrocytes and inflammatory exudates were detected in the pulmonary bronchi (Figure 12 and Figure 13).

## 4. Discussion

PRRS causes tremendous economic losses and remains a very important disease to manage in the global swine industry [36,37]. The high impact of PRRS has stimulated the development of MLV and KV vaccines to control the disease in breeding stock and nursery/growing pigs [15,16,17,18,19]. Several researchers have shown that MLV vaccines are more effective than KV ones, due to the stronger and more complete immune response that they can provide [15,16,18]. However, many concerns have been reported about their safety [15,16,17,19].

Based on our results, PRRSV-1 MLV vaccine strains could act as infectious strains when sows are vaccinated at the last stages of gestation, inducing congenital infections and affecting the immune status of newborn piglets. Several studies have also reported viremic newborn piglets derived from PRRSV-1 MLV-vaccinated gilts in the last trimester of gestation, supporting that the vaccination led to congenital infection and the PRRSV MLV virus was transmitted to fetuses [1,15,18,19]. Our present study indicates the passage of PRRSV-Abs into the circulation of congenitally infected piglets. Antibodies are not believed to be able to transfer to fetuses through the placenta due to their unique structural barriers. Hence, it is believed that the PRRSV-Abs response in fetuses is due to fetal infection [38,39]. Specifically, Butler and colleagues (2001) [38] and Rowland (2010) [39] suggested that maternally derived antibodies could enter the fetal circulation due to environmental antigens, which can cross the porcine placenta, similarly to PRRSV, and their subsequent de novo synthesis [28]. Thus, the above findings, in association with PRRSV viremia detected in several newborn piglets before the consumption of colostrum, explain the presence of PRRSV-Abs in the newborn piglets. Moreover, congenitally PRRSV-1 MLV-infected piglets display a poor clinical condition compared to the non-PRRSV-1 MLV-infected piglets.

Other researchers have reported porcine lungs as the primary site of PRRSV infection. In particular, the virus presents high tropism to alveolar pulmonary macrophages, in which it replicates initially, causing severe lesions in the pulmonary parenchyma. Subsequently, it spreads to other PRRSV-sensitive tissue (thymus, liver, spleen, lymph nodes, heart, and kidneys) [8,13,39]. Since data have been reported concerning lung lesions in transplacental infected newborn piglets, we decided, in the present study, to examine the lungs of euthanized piglets, to record potential lesions through macroscopical, histopathological, and SEM examination.

Necropsy revealed severe multifocal consolidation, interstitial edema, and scattered hemorrhages in RT-PCR (+) piglets. These findings are similar to those of Tian et al. (2007) [40], who studied a severe PRRS outbreak in weaning pigs that occurred in China and reported the presence of interstitial edema as well as hemorrhagic spots in the lungs. Furthermore, trials performed by Ming et al. (2017) [10] and Alpízar et al. (2018) [41] also described multifocal consolidation and hemorrhage in RT-PCR (+) 6- and 8-week-old pigs. In addition, Li et al. (2016) [42] exposed piglets aged 4 and 3 weeks, respectively, to PRRSV MLV vaccine strains and observed that they exhibited severe gross lesions with consolidation and hemorrhage. However, the difference between our research and the previous works lies in the age of the animals at the detection of pulmonary macroscopic lesions. Specifically, the present findings refer to newborn viremic piglets whose infection resulted from the PRRSV-1 MLV vaccination of gilts in late pregnancy and subsequent transplacental transmission.

On histopathological examination, interstitial pneumonia was detected in RT-PCR (+) piglets. Wang et al. (2017) [11] and Fan (2019) [9], describing the typical histopathological lung lesions of PRRSV-infected pigs, refer to the apoptosis of the pulmonary alveolar macrophages and the subsequent induction of inflammatory cell infiltration, resulting in interstitial pneumonia, supporting the histopathological lesions detected in the RT-PCR (+) piglets in our study. In addition, other researchers performed histopathological examinations on porcine lungs previously exposed to various PRRSV-1 MLV vaccine strains (3 weeks of age and older), detecting interstitial edema and diffuse thickening of the alveolar septa [10,41,42]. Furthermore, Gomez (2018) [43] reported, similarly to our study, the infiltration of lymphocytes, macrophages, and plasma cells in the alveolar septa of 4-week-old PRRSV-infected pigs. However, this study is the first one to investigate the vertical transmission and pathogenesis of the PRRS vaccine virus in neonatal piglets.

Moreover, in the histopathological examination of lung tissue samples (histopathological examination, examination under SEM), mild lesions were also found in RT-PCR (−) piglets, which belonged to litters from gilts that had been PRRSV-1 MLV-vaccinated on the 100th day of gestation. However, as revealed by the examination of RT-PCR (−) piglets belonging to the “control” group (no lesions were found), the presence of such lesions in a healthy organism is not justified. These findings may offer strong evidence that the virus, although it could not be detected, was transmitted vertically to the fetuses, causing mild lesions. Consequently, more research is needed to determine whether PRRSV MLV vaccine strains could act as a natural “wild” virus when administered at the end of pregnancy.

The lung tissue of RT-PCR (+) piglets was also tested under SEM. Their examination revealed accumulated proteinaceous and cellular exudates in the pulmonary alveolar spaces, leukocytes, lymphocytes, and macrophages, detected on histopathology. Pulmonary alveolar macrophages have been identified as the predominant cells supporting PRRSV replication [42]. In particular, the virus compromises its function and provokes inflammatory cell infiltration. As a result, high concentrations of leukocytes in the alveoli and bronchi are produced, and the presence of exudate is observed in the pulmonary parenchyma [44,45]. However, the SEM examination of lung tissue revealed the presence of additional lesions besides those described in the histopathological examination. Specifically, mild type II pneumocyte hyperplasia was detected histologically. This finding may be attributed to the cytopathic effect of the PRRSV-1 vaccine virus. PRRSV causes the degeneration of type I pneumocytes, and subsequent regeneration is achieved through type II pneumocyte hyperplasia [9,11]. Many researchers have recorded type II pneumocyte hyperplasia in PRRSV-infected pigs [10,41,42,43]. Therefore, previous trials confirm our findings from the examination of lung tissue under an optical microscope. Nevertheless, these researchers reported pulmonary lesions in pigs (3 weeks to 5 months old) challenged with a natural “wild” strain of PRRSV, whereas the present study revealed lesions in PRRS vaccine virus-infected newborn piglets.

Since the first description of PRRSV in the early 1990s, several researchers have investigated its etiology and pathogenesis. However, these have not yet been fully elucidated, and the virus continues to cause severe economic losses in the global pig industry [36,37]. Further macroscopic and histological examinations of lung tissue are commonly performed, and samples were observed in the present study to detect SEM lesions. Scanning and transmission electron microscopes, although not tools that are easily used in clinical practice and routine diagnostic procedures, have certain advantages over optical microscopes and can offer very important information to elucidate the mechanisms of action of the virus. In conclusion, electron microscopes could significantly enhance future researchers’ efforts to fully understand the etiology and pathogenesis of the virus.

## 5. Conclusions

Our study provides strong evidence that PRRSV-1 MLV vaccine strains can act as an infectious strain when vaccination is applied in the late stage of gestation, causing congenital infection and affecting the immune status of newborn piglets. The exposure of newborn piglets during their intrauterine life to a PRRSV-1 MLV strain negatively affected their respiratory systems, causing lesions of interstitial pneumonia. Therefore, our study highlights the serious risks that may arise from the late PRRSV-1 MLV vaccination of breeding stock. Based on our findings, during PRRSV-1 MLV mass vaccination, it is necessary to avoid the vaccination of pregnant gilts in the last two weeks before farrowing.

## Figures and Tables

**Table 1:** Descriptive statistics [mean] for all measured variables for each farm and group combination.

Group	Abortion Rate	Duration of Gestation (Days)	Number of Piglets					
			Live-Born	Dead-Born	Weak-Born	Mummies	Splay-Leg	With Respiratory Symptoms
**A**	0.0	118.4	16.0	1.4	0.8	0.0	0.0	0.0
**B**	0.0	117.4	13.4	1.8	1.6	0.4	0.0	0.8
**C**	0.0	114.2	9.8	2.2	3.0	2.0	1.2	2.4
**D**	0.0	116.8	8.8	1.6	1.6	0.4	0.4	1.0
**E**	0.0	119.2	15.0	1.8	2.0	0.0	0.0	0.6

**Table 2:** Estimates and 95% confidence intervals (CIs) for the effects of covariates on PRRSV-Abs levels.

Linear Regression			
Parameter	Hour after Bith	Estimate (95% CI)	*p*-Value
**Hour Birth**	0–3 h	4.3 (2.75; 6.71)	<0.001
	3–6 h	8.26 (5.77; 11.8)	<0.001
	6–9 h	26.33 (17.23; 40.24)	<0.001

## Data Availability

Not applicable.
